# Exploring the Efficacy of Various Minimally Invasive Surgery (MIS) Techniques in Spinal Fusion for Degenerative Spondylolisthesis: A Systematic Review

**DOI:** 10.7759/cureus.88723

**Published:** 2025-07-25

**Authors:** Yolanda V Gutierrez, Blake Martin, Joshua Nwose, Alyssa L Sepulveda, Ronald A Shaju, Aamir Ahmad, Robert Ablove

**Affiliations:** 1 Orthopedics, University of Texas Rio Grande Valley, Edinburg, USA; 2 Medicine, University of Texas Rio Grande Valley, Edinburg, USA; 3 Internal Medicine, University of Texas Rio Grande Valley, Edinburg, USA; 4 Orthopedic Surgery, University of Texas Rio Grande Valley, Edinburg, USA; 5 Orthopedic Surgery, Jacobs School of Medicine and Biomedical Sciences, Buffalo, USA

**Keywords:** degenerative spondylolisthesis, minimally invasive techniques, owestry disability index, spinal fusion, spine

## Abstract

Degenerative spondylolisthesis is a common etiology of low back pain characterized by the anterior displacement of one vertebral body relative to the one below it. A key contributing factor is intervertebral disc degeneration, which compromises spinal stability. In patients with a lack of neurological issues, conservative treatment is recommended. If conservative treatment is ineffective, surgery is deemed the next step, involving decompression and fusion of the vertebrae. Over the past several decades, minimally invasive surgical (MIS) techniques have been developed and refined. This systematic review aims to compare different types of MIS techniques for the treatment of degenerative spondylolisthesis to determine the advantages and disadvantages of each.

We searched PubMed and manually screened The Global Spine Journal and The Journal of Orthopaedic Surgery and Research using Boolean operators to identify studies published from 2015 to 2025. Inclusion criteria encompassed retrospective cohorts, randomized controlled trials, and comparative studies of patients with degenerative spondylolisthesis. Risk of bias was qualitatively assessed based on study design and reporting. Due to heterogeneity, data were synthesized descriptively.

A total of 302 studies were initially identified; only 13 met the inclusion criteria. A total of 714 patients were included in the review. The most common technique was MIS transforaminal lumbar interbody fusion (TLIF), followed by MIS OLIF. All the studies demonstrated improvement in the Oswestry Disability Index (ODI) post-operatively. Secondary outcomes such as operation time, blood loss, radiographic fusion rates, and hospital stay varied across the studies.

Both MIS TLIF and MIS OLIF have demonstrated effectiveness in the surgical management of degenerative spondylolisthesis, with MIS TLIF showing favorable outcomes for single-level decompression and MIS OLIF for multilevel fusion and deformity correction. However, variability in follow-up duration, outcome reporting, and patient selection limits direct comparison. While MIS approaches may reduce perioperative morbidity compared to traditional open fusion and can be effective after failed conservative treatment, current evidence does not definitively favor one technique over the other. Further high-quality, comparative studies are needed to establish superiority in terms of long-term outcomes and complication rates.

## Introduction and background

Spine diseases and degeneration are common causes of back pain in the population; common spinal conditions include disc degeneration, disc herniation, dysarthrosis, spinal canal stenosis, and facet joint arthrosis [1]. Alongside these causes of back pain, spondylolisthesis, which is the slipping of a vertebral body or disc, is also common [2]. Six subtypes of spondylolisthesis exist: congenital (dysplastic), isthmic, degenerative, traumatic, pathological, and postsurgical. This literature review will focus specifically on degenerative spondylolisthesis and the effectiveness of various minimally invasive surgical (MIS) techniques currently available.

Degenerative spondylolisthesis often occurs due to the breakdown of the intervertebral disc, which causes spinal instability. The prevalence of this type of spondylolisthesis varies by age and gender, being more common in the elderly and women, with a ratio of 5:1 compared to men [3]. Other risk factors include longer driving hours, manual labor, and increased sedentary activity [4]. In the past, spondylolisthesis was primarily treated in patients with spondylolysis, a condition that usually affects young individuals [5]. Recently, there has been progress in understanding the disease among older adults, emphasizing the identification of degenerative spondylolisthesis [5]. Advances in technology and surgical techniques, along with better supportive care, now offer spine surgeons more effective treatment options [5]. In patients without neurological issues, conservative management is recommended [6]. If patients continue to experience persistent or worsening symptoms - such as back or leg pain, functional impairment, or neurological deficits - despite an adequate trial of conservative treatment, surgical intervention, typically involving decompression and spinal fusion, is considered the next step [7]. Over the past decade, MIS techniques have emerged, showing benefits such as reduced blood loss, lower hospitalization rates, and less damage to surrounding tissues [8]. Although relatively new, recent studies have shown promising results.

Due to the short time MIS techniques have been implemented, reviewing literature to determine their efficacy will further benefit patients and the orthopedic surgery community. This systematic review focuses solely on patients with degenerative spondylolisthesis undergoing MIS spinal fusion techniques, and differing MIS techniques are compared to each other with the extraction of the outcomes such as the Oswestry disability index (ODI), radiographic fusion, blood loss, and more. The objective of this systematic review is to compare the clinical outcomes and complications of different MIS techniques used for spinal fusion in patients with degenerative spondylolisthesis.

Overview of treatment for degenerative spondylolisthesis

The treatment for degenerative spondylolisthesis is usually conservative. In cases where there is no spinal nerve compression, non-surgical methods are highly recommended, which include a combination of physiotherapy, anti-inflammatory medication, control of body weight, and medication for osteoporosis [9-11]. This regimen has been utilized for more than 20 years and is still used to this day [10]. However, when neurological symptoms begin to occur, surgical interventions such as posterior lumbar interbody fusion (PLIF), transforaminal lumbar interbody fusion (TLIF), and lateral lumbar interbody fusion (LLIF) could be recommended [11]. Although there are advantages and challenges for each type of surgery, surgical treatment is ultimately individualized based on patients’ health status [11]. According to the Spine Patient Outcome Research Trial (SPORT), usually, the best line of treatment for patients is surgery instead of conservative management [11]. This was determined by comparing patients who participated in surgery and patients who were undergoing conservative treatment and were followed for 2, 4, and 8 years; the results demonstrated that patients who underwent surgery had greater improvement of symptoms [12]. In turn, surgery possesses a beneficial outcome for patients experiencing chronic pain that has not been relieved with conservative management.

Although many types of surgical procedures can be done based on the complication the patient might have, the first procedure to be explored for degenerative spondylolisthesis is the posterior interbody lumbar fusion, which was being investigated mainly for its ability to decompress the spinal nerves and relieve neurological symptoms that could occur with severe degenerative spondylolisthesis [13,14]. Previously, Cloward states other techniques were investigated for lumbar disease, such as simple discectomy, decompressive laminectomy, and chemonucleolysis; ultimately, PLIF was found to be the best course of surgical treatment [15]. Along with these procedures, anterior lumbar interbody fusion (ALIF) is a highly recommended procedure in the realm of degenerative spondylolisthesis. ALIF is highly advantageous compared to PLIF and TLIF for various reasons. Some benefits of ALIF are the ability to accommodate larger implants due to better surgical visibility, increased fusion outcomes, and decreased disruption to the muscles upon surgical incision [16-18]. Although there are increased benefits for this procedure, there are also risks and contraindications. ALIF is contraindicated in various situations such as recent abdominal surgery, spine infections, peripheral vascular disease, and more [18]. Another procedure that has been acknowledged for its ability to minimize blood loss is LLIF, which has also been adapted to another procedure called extreme lateral interbody fusion (XLIF). This procedure can be helpful when ALIF is contraindicated for patients who might have previous abdominal procedures; however, this procedure can only be used to address discs T12/L1 - L4/L5 due to the probability of injuring major blood vessels below L5 [11,19]. The alternative XLIF provides very similar benefits, with the major benefit being that it is more minimally invasive [19]. It was also found that due to the minimally invasive techniques of XLIF, there were lower infection rates, decreased risk of injury to the spinal cord, and shorter hospitalization [20]. Although these techniques have been improved with increased benefits for patients, there is now an exploration of further increasing these techniques with MIS procedures.

MIS techniques have recently been implemented in many types of surgeries. And now, recently been implemented in spinal fusion surgeries for degenerative spondylolisthesis. The increased alleged benefits of MIS techniques lie in the decreased blood loss, lower hospitalization rates, and fewer complications during surgery [21]. Although MIS techniques have increased benefits, they also have certain disadvantages. Since these techniques are new, there is the disadvantage of needing training and experience to be able to perform a successful procedure [21,22]. Comparing and contrasting different MIS procedures could be helpful for the orthopedic surgery community to guide treatment options. A portion of this article was previously presented as a poster at the 2024 UTRGV Research Colloquium on September 13th, 2024.

## Review

Methods and materials

Data Collection

This systematic review was conducted and reported in accordance with the Preferred Reporting Items for Systematic Reviews and Meta-Analyses (PRISMA)[23]. This study was IRB-exempt as the data were publicly available. The objective of this review was to compare different MIS techniques for the treatment of degenerative spondylolisthesis (DS), with outcome measures including the Oswestry Disability Index (ODI), estimated blood loss, operative time, hospital stay, and radiographic fusion rates. Five reviewers (YG, BM, JN, AS, and RS) independently screened titles and abstracts using the defined inclusion criteria. Full texts of eligible studies were then reviewed in detail. Data extraction was performed independently, and any discrepancies were resolved by discussion and consensus among the authors. Extracted data included: Oswestry disability index, Fusion outcomes, operative time, estimated blood loss, and length of hospital stay.

Study Criteria

Studies were eligible if they were randomized controlled trials, retrospective cohort studies, or comparative studies that focused on patients with DS undergoing MIS spinal fusion. For mixed population studies, only those that were stratified for outcomes for DS patients were included. Studies were excluded if they were non-English, animal studies, cadaveric studies, meta-analyses, systematic reviews, or lacked stratification by DS.

Study Design

The initial search began in June 2024 and ended in May 2025. We conducted a formal database search using PubMed, and also performed manual searches of The Global Spine Journal and The Journal of Orthopaedic Surgery and Research. The same Boolean search strategy was applied across all sources using the terms:

((spondylolisthesis) AND (degenerative)) AND (minimally invasive). Filters included English language, human studies, and publication dates between 2015 and 2025 to capture the most recent advancements in MIS techniques. The primary outcome assessed was the ODI, which quantitatively measures functional improvement after surgery. Secondary outcomes included radiographic fusion, operation time, estimated blood loss, and average hospital stay.

Results

Study Characteristics

Most studies were retrospective cohorts, with a minority being prospective cohorts or randomized controlled trials. A total of 302 studies were initially identified through database and journal searches. After the removal of duplicates, 43 full-text articles were reviewed. From these articles, only 13 studies met the inclusion criteria and were included in the final analysis.

Reasons for the exclusion of certain articles included: lack of ODI data, lack of preoperative ODI data, and mixed studies that did not stratify data based on DS but instead grouped many degenerative spine disorders.

Across all studies, there is a total of 714 patients, with the most common MIS being TLIF. All the studies that were included specifically involved patients with DS who underwent spinal fusion. Although spinal fusion was the treatment for the patients, varying techniques were involved. The procedures of the studies involved include MIS TLIF, MIS OLIF, MIS PLIF, and MIS XLIF.

Data was extracted independently by five researchers (YG, BM, JN, AS, and RS). Discrepancies were resolved through discussion and consensus. No automation tools were used in the selection process. Risk of bias was assessed narratively based on study design, sample size, reporting clarity, and potential confounders. A formal risk of bias tool was not applied due to the variability in study designs; however, a narrative appraisal of study quality was conducted, considering potential sources of bias such as selection, performance, and reporting bias.

**Figure 1:**
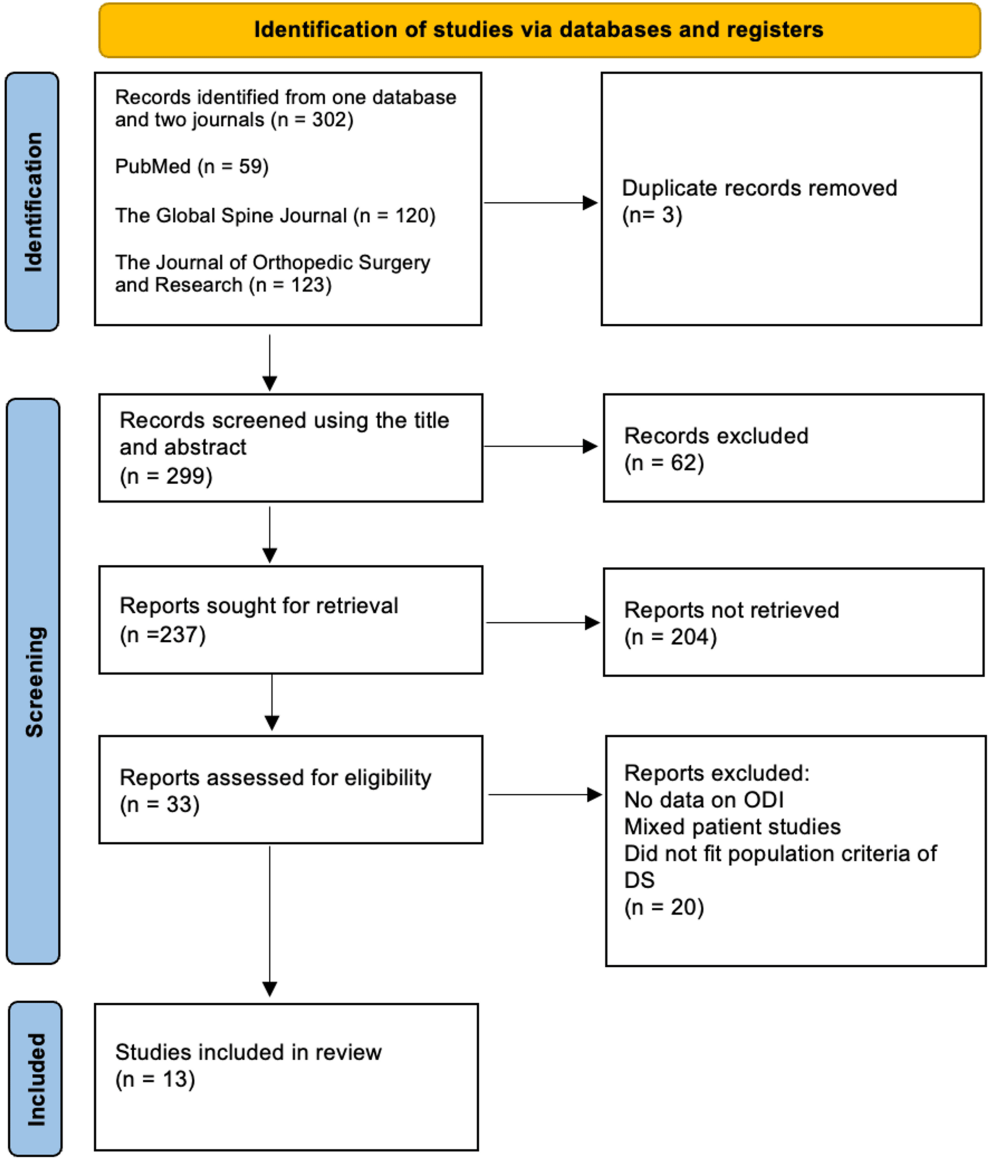
2020 PRISMA Flowchart for Systematic Review of MIS Techniques for Degenerative Spondylolisthesis. MIS: minimally invasive surgery

Due to the clinical methodology and heterogeneity among the included studies, such as differences in study design, patient populations, interventions, and outcome measures, a meta-analysis was not possible. Therefore, a narrative synthesis was conducted. The data was extracted systematically and synthesized descriptively to identify common patterns or variations. This approach allowed for a structured comparison of results while accounting for variations across the studies. Table 1 provides a summary of the characteristics and outcomes of the included studies. Figure 1 depicts the PRISMA flowchart of the studies included in [23].

**Table 1:** Summary of article data extraction results for MIS techniques in degenerative spondylolisthesis MIS TLIF: Minimally invasive transforaminal lumbar interbody fusion; OLIF: oblique lateral interbody fusion; ODI: Oswestry Disability Index; PPS: percutaneous pedicle screw

Article	Study Type	Procedure Type	Patient Number (n)	ODI	Radiograph Fusion Rates	Operation time (min.)	Estimated Blood Loss (mL)	Average Hospital Stay (Days)
Ali et al. (2024)[24]	Retrospective comparative	MIS TLIF	60	Preop: 48.7 ± 6.6 1 Month, Post-op: 21.4 ± 2.4 6 Months, Post-op: 21.5 ± 2.5 24 Months, Post-op: 21.6 ± 2.3	Not reported	134.8 ± 26.1	256 ± 139	3.2 ± 0.9
Bahir et al. (2024)[25]	Retrospective comparative	MIS TLIF	45	Preop: 58.37 ± 6.01 3 days, post-op: 37.84 ± 6.16 3 months, post-op: 27.26 ± 5.81 12 months, post-op: 19.66 ± 4.43 2 years, post-op: 13.11 ± 2.56	97.7% after 2 years	146.51 ± 13.25	200.88 ± 25.86	9.08 ± 1.42
Mohanty et al. (2025) [26]	Retrospective matched cohort study	MIS TLIF	52	Preoperative: 36.62 ± 0.98 6 weeks, post-op: 30.61± 1.26, 1-year post-op: 18.31± 1.44	Not reported	Not reported	Not reported	Not reported
Le et al. (2021)[27]	Retrospective matched cohort	MIS TLIF	38	Change in ODI: 7	Not reported	310.8	282.4	6.4
Chan et al. (2019)[28]	Prospective, multicenter cohort	MIS TLIF	72	Preop: 46.2 ± 16.3, Post-op at 24 months: 14.3 ± 17.2	Not reported	228.2 ± 111.5	108.8 ± 85.6	2.9 ± 1.8
Wu et al. (2019) [29]	Retrospective comparative	MIS OLIF MIS PLIF	31 47	Preoperative: 59.7±6.3, Post-op: 25.1±4.9, Preoperative: 59.1±6.8, Post-op: 28.5±7.7	Not reported	131.3±14.6 156.9±37.4	163.6±63.9 496.8±122.6	Not reported
Kim et al. (2015) [30]	Retrospective cohort study	MIS TLIF	23	Preop: 45.8, 1-month post-op: 24.2, 6-month post-op: 18.1, 1-year post-op: 15	87%	120.78±20.51	203.91±159.93	7.86±2.36
Coric et al. (2023) [31]	Prospective randomized controlled trial	MIS TLIF	79	Preoperative: 56.4±13.3, 6-week post-op: 30.6, 24-month post-op: 36.2	Not reported	178.1±58.7	223.1±133.3	2.95±1.85
Han et al. (2021) [32]	Retrospective cohort	MIS TLIF	33	Preoperative: 46.4±16.1, 3-month post-op: 26.1±12.8	87%	121.5±48.2	291.5±72.3	4.2±2.5
Ohba et al. (2017) [33]	Prospective comparative cohort	MIS XLIF and PPS	46	Preoperative: 21.2±6.9, 1-year post-op: 9.2±7.4	100%	Not reported	51±41	7
Qin et al. (2020)[34]	Retrospective Cohort	MIS TLIF	34	Preoperative: 42.34±4.61, 3-month post-op: 21.93±3.58, 6-month post-op: 17.27±2.47, 12-month post-op: 15.88±2.07, last follow-up: 14.71±1.21	100%	143.94±11.59	170.56±11.98	10.26±1.35
Li et al. (2021) [35]	Retrospective comparative study	MIS TLIF MIS OLIF	35 28	Preop: 53.93, Preop: 54.88±8.13 6.06 1-week post-op: 28.13±2.07, 3-month post-op: 18±1.77, Preop: 54.88±8.13, 1-week post-op: 22.44±2.61, 3-month post-op: 17.06±1.29	Not reported	199±59.64 186.44±36.5	190±66.33 55.94±57.37	12.87±2.60 7.06 ±2.51
Mummaneni et al. (2017) [36]	Retrospective registry-based cohort	MIS TLIF	91	(change in ODI) Level 1: -27.61 Level 2: -27.43	Not reported	Level 1: 212 Level 2: 282	Level 1: 143 Level 2: 220	Level 1: 3.21 Level 2: 4

Outcomes

All 13 studies included the ODI preoperative and postoperative results. However, due to different studies, many report scores at different times of follow-up, while some only report one follow-up score. All the studies showed consistent improvement in the ODI scores. The highest preoperative value was observed in an MIS OLIF study at 59.7±6.3 [29]. The lowest postoperative score was observed in an MIS XLIF and percutaneous pedicle screw (PPS) study [33] at 9.2±7.4 one-year post-op. Two studies didn’t report exact ODI values but reported the difference between preoperative ODI and postoperative ODI being: Level 1: -25.54 and Level 2: -27.43 [36], and seven in Le et al. (2020) [28].

Secondary outcomes included radiographic fusion rates, estimated blood loss, operative time, and length of hospital stay. Radiographic fusion rates between the 13 studies included ranged between 100% to 87%. Operative time had the lowest time at 120.78±20.51 minutes in an MIS TLIF procedure [31] and the highest time of 310.8 minutes in an MIS TLIF procedure [28]. Estimated blood loss was the lowest in an XLIF and PPS procedure at 51±41 mL [33] and highest in an MIS PLIF at 496.8±122.6 [30]. Lastly, the length of stay in the hospital was the lowest in an MIS TLIF procedure at 2.9 ± 1.8 days [28], and the longest stay was observed in another MIS TLIF study at 12.87±2.60 [36].

Discussion

MIS techniques have demonstrated a range of benefits. Some of them include decreased blood loss, minimized post-surgery pain, and shorter recovery time [37]. A study determined that Thoraco-lumbar posterior fusion therapy was causing patients severe constipation and vomiting, possibly due to increased medication during and after a long surgery [38]. It was concluded that constipation is highly associated with longer operation times, high blood loss, and increased administration of morphine for pain; therefore, MIS techniques could benefit these patients and prevent further side effects that are seen in OS [38].

Efficacy of MIS techniques for spinal fusion can be evaluated in a variety of ways. Some of the most common ways to evaluate efficacy are determining the ODI, recovery times, and patient satisfaction. Comparing these values could help the research community observe the advantages and disadvantages of using different MIS techniques. Another measure that could be used to determine the efficacy of MIS techniques is the length of stay in the hospital for recovery, estimated blood loss, and fusion rates.

Among the studies included, the most common procedure conducted was MIS TLIF, with 11 out of 13 studies. The next common procedure conducted was MIS OLIF, with 2 out of 13 studies. Current literature demonstrates that no procedure is superior to another. Each procedure has its advantages and disadvantages. While MIS TLIF was the most common procedure reported in this review, its selection may be influenced by factors such as patient anatomy, surgeon experience, or procedural familiarity. Although MIS techniques overall are associated with lower complication rates compared to open surgery, certain patient populations or surgical goals may influence the choice of one MIS technique over another due to differences in anatomical access, operative time, or technical complexity. Current literature demonstrates that this procedure is preferred to others, such as MIS OLIF, for its ability to provide better results for direct decompression [39]. MIS TLIF has demonstrated decreased blood loss, shorter hospital stays, and fewer complications, but it has been shown to have decreased efficacy in patients needing a durotomy [39-41]. On the other hand, MIS OLIF has been more commonly used to treat patients with degenerative scoliosis and other degenerative diseases that require multilevel spinal fusions due to its ability to provide better exposure of the intervertebral disc region during the surgery [42]. Some complications that the MIS OLIF procedure is associated with are vascular and sympathetic damage [16,43]. However, it’s important to note that in this review, many of the MIS TLIF studies demonstrated higher rates of blood loss and operation time compared to other procedures. This variability in operation results could be a result of many variables, such as surgical complications or surgeon techniques. Therefore, it is important to analyze each case individually and consider the variability.

A major strength of this review is the strict inclusion criteria. This selective criterion allowed us to observe results in a specific population: DS. The removal of mixed studies that did not separate results based on the type of degenerative lumbar diseases also makes the study more specific to the population being studied. Our decision to only include studies that were published in the last 10 years also allowed us to only compare the most recent studies to stay up to date with novel technology and techniques. To our knowledge, this is the first systematic review to compare different MIS spinal fusion techniques exclusively in patients with DS.

Although this review demonstrates strengths, limitations must be noted as well. Although all the studies included in this review have exact ODI values, many studies had many follow-up values, while some only had preoperative and postoperative values. Other limitations of the review process include a lack of protocol registration and the absence of a formal risk of bias assessment tool. Additionally, the inability to perform a meta-analysis limits the statistical strength of the synthesis.

There is still no common consensus on whether there is a specific MIS procedure that is superior to another for the treatment of DS. However, the studies included in this review demonstrated greater operative times and length of hospital stay compared to other techniques in MIS TLIF. Although there are varying changes between the ODI in different types of procedures, all the studies demonstrated significant improvement in the ODI. With these results, it can be concluded that the type of procedure for a patient should be determined on a case-by-case basis; even in patients with DS, such as patients requiring multilevel fusion, might benefit from MIS OLIF over other techniques.

## Conclusions

DS is a common, age-related spinal condition that frequently affects older adults and individuals with a sedentary lifestyle. With continued advancements in surgical technology, MIS techniques have become increasingly utilized. These approaches offer significant benefits, including reduced blood loss, shorter hospital stays, and faster recovery. Such advantages may improve surgical outcomes and quality of life for patients undergoing treatment for DS. Although multiple MIS techniques are available, no single method has shown clear superiority. Therefore, surgical decision-making should be individualized based on the patient’s specific clinical presentation, anatomical factors, and treatment goals.
